# The interplay between *GBA1* status and age of onset on cognitive, motor and non-motor outcomes in Parkinson’s disease: multicenter cross-sectional study

**DOI:** 10.1007/s00415-026-13639-x

**Published:** 2026-02-01

**Authors:** Claudia Ledda, Silvia Gallo, Micol Avenali, Carlo Alberto Artusi, Gabriele Imbalzano, Francesca Donetto, Elisa Montanaro, Alberto Romagnolo, Pierfrancesco Mitrotti, Luca Gallo, Rosa De Micco, Valeria Sant’Elia, Mattia Siciliano, Alessandro Tessitore, Giovanna Calandra-Buonaura, Giulia Giannini, Luisa Sambati, Leonardo Lopiano, Enza Maria Valente, Marco Bozzali

**Affiliations:** 1 Department of Neuroscience “Rita Levi Montalcini”, University of Turin, Via Cherasco 15, 10126 Turin, Italy; 2 SC Neurologia 2U, AOU Città della Salute e della Scienza, Turin, Italy; 3 Department of Brain and Behavioral Sciences, University of Pavia, Pavia, Italy; 4 IRCCS Mondino Foundation, Pavia, Italy; 5 Department of Advanced Medical and Surgical Sciences, University of Campania “Luigi Vanvitelli”, Naples, Italy; 6 Department of Psychology, University of Campania Luigi Vanvitelli, Caserta, Italy; 7 IRCCS Istituto delle Scienze Neurologiche di Bologna, Bologna, Italy; 8 Department of Biomedical and NeuroMotor Sciences, Alma Mater Studiorum, University of Bologna, Bologna, Italy; 9 Department of Molecular Medicine, University of Pavia, Pavia, Italy

**Keywords:** Age at onset, Parkinson’s disease, *GBA1*, Non-motor symptoms, Cognitive decline

## Abstract

**Background and objectives:**

Age at onset is a key determinant of disease course in the general Parkinson’s disease (PD) population, but its influence among GBA-PD remains undetermined. This study investigates whether age at onset affects cognitive decline in GBA-PD patients and compares symptoms between GBA-PD and nonGBA-PD groups, stratified by age of onset.

**Methods:**

In this multicentric cross-sectional study, PD patients were stratified into early onset (< 50 years), intermediate onset (50–60 years), and late onset (> 60 years). Demographic–clinical data and scores of the Movement Disorder Society—Unified Parkinson’s Disease Rating Scale (MDS-UPDRS), Montreal Cognitive Assessment (MoCA), Scales for Outcomes in Parkinson’s Disease—Autonomic Dysfunction (SCOPA-AUT), and Beck Depression Inventory (BDI-II) were compared using ANCOVA. The effects of age of onset, *GBA1* status, and their interaction were investigated. External validation on cognition was performed using data from the PPMI cohort.

**Results:**

We analyzed 80 GBA-PD and 236 nonGBA-PD patients. Among GBA-PD, late-onset patients exhibited worse axial scores (*p* = 0.037), while early-onset had more severe motor complications (*p* = 0.007) and dysautonomia (*p* = 0.012). Age of onset and *GBA1* status did not influence MoCA scores. Conversely, *GBA1* status independently affected MDS-UPDRS parts I and II (*p* < 0.001 and *p* = 0.019, respectively) and BDI-II scores (*p* = 0.002). Analysis on the external dataset (PPMI) showed late-onset PD had lower MoCA scores (*p* < 0.001) and confirmed *GBA1* status did not influence cognition.

**Discussion:**

In the first decade of PD, cognitive decline is mainly age and duration dependent, irrespective of *GBA1* genotype. Early onset does not increase cognitive risk in GBA-PD, supporting its relevance for counseling and treatment planning.

**Supplementary Information:**

The online version contains supplementary material available at 10.1007/s00415-026-13639-x.

## Introduction

*GBA1* variants are the most frequent genetic risk factor for the development of Parkinson’s disease (PD) [1–4]. Indeed, heterozygous variants in the *GBA1* gene have been identified in 10–15% of PD patients, depending on the population under study [2, 3, 5]. While patients with and without *GBA1* variants can be indistinguishable at an individual level, those with *GBA1* variants (GBA-PD) collectively show earlier disease onset, faster motor decline, higher burden of non-motor symptoms, increased risk of mortality, and, importantly, accelerated cognitive decline [1, 2, 6–11]. These findings have raised important questions about whether *GBA1* carriers require distinct management approaches and whether certain therapies, such as deep brain stimulation (DBS), are suitable for these patients [12–15].

For idiopathic PD, numerous studies have demonstrated that the younger age of disease onset influences specific clinical features, including a lower risk of cognitive decline, a higher burden of fluctuations and dyskinesia, reduced quality of life, and slower disease progression [16–19]. However, the impact of age at disease onset in the GBA-PD population has been overlooked, and it remains unclear whether an earlier onset is associated with a more severe or milder phenotype in terms of both motor and non-motor (e.g., cognitive, neuropsychiatric, autonomic) symptoms.

In this context, the primary aim of this study was to investigate whether an earlier age at onset in GBA-PD patients is a risk factor for worse cognitive functioning. The secondary aim was to assess whether motor and non-motor symptoms differ between *GBA1* carriers and non-carriers, stratified for age of disease onset.

## Methods

This is a multicentric cross-sectional study, involving four Movement Disorder Centers in Italy (Department of Neurosciences “Rita Levi Montalcini”, University of Turin; Department of Advanced Medical and Surgical Sciences, University of Campania “Luigi Vanvitelli”; Parkinson and Movement Disorders Unit, IRCCS Mondino Foundation, of Pavia; IRCCS Istituto delle Scienze Neurologiche of Bologna). The study was conducted according to the STROBE guidelines for observational studies [20].

A multicentric cohort of patients with available *GBA1* genotype was recruited in the period from May 2023 up to October 2024. The *GBA1* gene was tested using a novel NGS-based method, which relies on the selective amplification of the whole gene in one long PCR fragment (6 kb) followed by Nextera sequencing and a customized bioinformatics pipeline aimed at masking the *GBAP1* pseudogene [21].

In addition, all patients with onset < 50 years or positive family history for PD were tested with a next-generation sequencing (NGS) panel targeting major genes associated with parkinsonism (*ATP13A2*, *C19orf12*, *DJ1*, *FBXO7*, *HGSNAT*, *LRRK2*, *PINK1*, *PLA2G6*, *POLG*, *PRKN*, *SLC6A3*, *SNCA*, *TAF1*, *VPS35*).

Inclusion criteria included a PD diagnosis, as per the Movement Disorder Society (MDS) diagnostic criteria [22], and age ranging from 18 to 80 years. Exclusion criteria were the presence of atypical parkinsonism or other neurological disorders, the presence of pathogenic or likely pathogenic variants in PD-related genes other than *GBA1*, and treatment with device-aided therapies (i.e., infusion therapies or deep brain stimulation).

### Standard protocol approvals, registrations, and patient consents

The Local Ethics Committee approved the study in each center (protocol number 0044208), and all participants provided written informed consent.

### Outcome measures

We collected clinical and demographical data, including age, sex, disease duration, years of formal education, and dopaminergic therapy, with levodopa equivalent daily dose (LEDD) calculated using a validated formula [23]. Non-motor and motor experience of daily living activities (ADL) were assessed using the Movement Disorder Society-sponsored Unified Parkinson’s Disease Rating Scale (MDS-UPDRS) part I and II, respectively [24], while motor impairment was evaluated by means of the MDS-UPDRS part III, carried out in the daily on condition. The axial score was calculated by summing the following items of the MDS-UPDRS part III: item 3.1 (speech), 3.3a (neck rigidity), 3.9 (arising from a chair), 3.10 (gait), 3.11 (freezing of gait), 3.12 (postural stability) and 3.13 (posture) [25]. Motor complications (MC) (including both dyskinesia and motor fluctuations) were evaluated by means of the MDS-UPDRS part IV [24], global cognitive status with the adjusted Montreal Cognitive Assessment (MoCA) [26], autonomic dysfunction by means of the Scales for Outcomes in Parkinson’s disease—Autonomic Dysfunction [27], and depression with the Beck Depression Inventory (BDI-II) [28].

Patients were categorized as *GBA1* carriers (GBA-PD) or non-carriers (nonGBA-PD) and stratified for age of onset of motor symptoms in three groups: early-onset PD (EOPD; onset < 50 years of age), intermediate-onset PD (IOPD; onset ≥ 50 and ≤ 60 years of age), and late-onset PD (LOPD; onset > 60 years of age) [29]. The categorization into normal cognition, mild cognitive impairment (MCI) or dementia was performed for each group by validated cut-offs of the adjusted MoCA score [30].

### Statistical analysis

Descriptive statistics was used for continuous variables, while frequency was used for categorical data. Analysis of covariance (ANCOVA) was conducted to compare continuous outcomes across three age-at-onset groups within the GBA-PD and nonGBA-PD cohorts, adjusting for disease duration. In addition, a two-way ANCOVA was used to assess the main effects of age at onset and *GBA1* status, as well as their interaction, while controlling for disease duration as a covariate. The same analysis was repeated for MoCA scores adjusted for age at assessment and education, following the established criteria [31]. Statistical analyses were corrected for multiple comparisons using Bonferroni’s adjustment.

A Chi-square test was used to compare the distribution of normal cognition, MCI, and dementia between GBA-PD and nonGBA-PD patients within each age-at-onset category (EOPD, IOPD, LOPD).

Finally, to validate our findings, we analyzed the effect of age of onset and *GBA1* status on the MoCA score using data from the Parkinson’s Progression Markers Initiative (PPMI) cohort, a multicenter longitudinal study collecting clinical information from a large cohort of individuals with PD [32]. We included all PD patients from the PPMI dataset with a positive dopamine transporter single-photon emission computed tomography (DaT-SPECT), who had undergone genetic analysis and had complete clinical data, including MoCA scores, available 4–6 years after disease onset. A follow-up time point of 4 to 6 years from the clinical onset of PD was chosen to ensure both a sufficiently large patient population and a follow-up duration long enough to capture meaningful disease progression.

Data with > 30% of missing values were excluded from the analyses.

All the analyses were performed with Statistical Package for the Social Sciences (SPSS 27.0 for Macintosh, Chicago, IL), using two-tailed p-values with a level of significance of 0.05.

### Data availability

The data that support the findings of this study are available from the corresponding author upon reasonable request.

## Results

A total of 382 patients with a clinical diagnosis of PD were screened for eligibility across participating centers. Of these, 66 were excluded for the following reasons: 23 declined or did not complete genetic testing, 17 had incomplete clinical or cognitive data, 12 were receiving advanced PD therapies, 8 presented with other neurological or cerebrovascular conditions that could significantly affect cognitive performance (such as extensive small-vessel disease, prior stroke, or comorbid neurodegenerative disorders), and 6 did not meet inclusion criteria due to uncertain diagnosis.

The final cohort thus included 316 PD patients who were included in the study (197 males and 119 females), of whom 80 were GBA-PD. For cohort stratification based on age at onset, *GBA* status, and *GBA* variants, see Supplementary Table 1.

Among GBA-PD patients, 51% were EOPD (41/80), 34% IOPD (27/80), and 15% LOPD (12/80). Among nonGBA-PD patients, 17% were EOPD (40/236), 46% IOPD (109/236), and 37% LOPD (87/236). Clinical and demographic data of the entire cohort and divided per age of onset groups are reported in Table 1. Classification of patients into normal cognition, MCI and dementia stratified per age of onset and *GBA1* status are reported in Table 2.

**Table 1:** Clinical and demographical characteristics of nonGBA-PD and GBA-PD patients stratified by age group

	EOPD	IOPD	LOPD	*p*-value (ANCOVA)
Sex %M/%F (M/F)				
GBA-PD	58.5%/41.5% (24/17)	59.3%/40.7% (16/11)	50%/50% (6/6)	NA
nonGBA-PD	60%/40% (24/16)	70.6%/29.4% (77/32)	57.5%/42.5% (50/37)	NA
Age at evaluation				
GBA-PD	51.63 ± 6.51 (37–65)	61.37 ± 4.29 (52–71)	72.58 ± 4.70 (67–82)	< 0.001*
nonGBA-PD	51.88 ± 7.79 (36–68)	63.29 ± 4.06 (53–79)	71.84 ± 4.87 (61–89)	< 0.001*
Age of onset				
GBA-PD	41.51 ± 5.5 (27–49)	55.22 ± 3.11 (50–60)	66.17 ± 4.84 (61–74)	< 0.001*
nonGBA-PD	43.28 ± 5.07 (28–49)	55.34 ± 3.1 (50–60)	66.76 ± 4.57 (61–81)	< 0.001*
Disease duration				
GBA-PD	10.15 ± 6.04 (3–26)	6.19 ± 3.46 (2–15)	6.42 ± 1.93 (3–10)	0.003*
nonGBA-PD	8.75 ± 5 (2–26)	7.94 ± 3.59 (1–22)	5.15 ± 2.94 (1–15)	< 0.001*
LEDD				
GBA-PD	836.68 ± 528.11 (200–3300)	714.78 ± 493.16 (0–1997)	693.42 ± 403.12 (200–1564)	0.672
nonGBA-PD	863.70 ± 585.06 (0–2105)	796.87 ± 475.27 (0–2543)	490.45 ± 295.68 (0–1725)	< 0.001*
Education				
GBA-PD	13.34 ± 3.27 (8–20)	11.85 ± 3.98 (5–19)	9.92 ± 5.11 (5–19)	0.011*
nonGBA-PD	12.56 ± 3.43 (5–18)	11.60 ± 3.81 (5–22)	10.93 ± 4.36 (5–19)	0.188
MDS-UPDRS I				
GBA-PD	10.67 ± 6.38 (2–26)	11.56 ± 3.59 (4–29)	9.67 ± 5.90 (2–18)	0.427
nonGBA-PD	8.60 ± 6.3 (0–22)	7.69 ± 5.89 (0–24)	5.48 ± 5.86 (0–24)	< 0.001*
MDS-UPDRS II				
GBA-PD	11.38 ± 6.99 (0–29)	12.41 ± 6.93 (2–27)	9.75 ± 5.85 (0–18)	0.067
nonGBA-PD	10.42 ± 7.7 (0–31)	9.27 ± 7.18 (0–39)	6.62 ± 6.63 (0–30)	< 0.001*
MDS-UPDRS III				
GBA-PD	23.50 ± 15.73 (6–57)	25.74 ± 12.67 (2–48)	26.83 ± 13.5 (4–49)	0.194
nonGBA-PD	24.18 ± 13.37 (2–66)	26.09 ± 13 (4–71)	27.31 ± 11.95 (5–58)	0.140
MDS-UPDRS IV				
GBA-PD	5.03 ± 4.64 (0–15)	4.42 ± 4.16 (0–16)	2.67 ± 3.06 (0–10)	0.007*
nonGBA-PD	6.54 ± 3.96 (0–13)	4.57 ± 4.79 (0–17)	1.43 ± 2.36 (0–12)	< 0.001*
Axial Score				
GBA-PD	4.85 ± 4.1 (0–18)	5.19 ± 2.76 (0–10)	5.82 ± 4.58 (1–15)	0.037*
nonGBA-PD	4.93 ± 4.5 (0–23)	5.23 ± 3.63 (0–23)	5.90 ± 3.27 (0–18)	< 0.001*
SCOPA-AUT				
GBA-PD	14.88 ± 9.28 (2–38)	14.00 ± 6.47 (2–30)	14.80 ± 8.53 (1–29)	0.012*
nonGBA-PD	14.89 ± 9.13 (0–32)	14.55 ± 8.56 (0–49)	14.10 ± 8.6 (0–49)	0.244
BDI-II				
GBA-PD	13.00 ± 10.56 (1–63)	10.75 ± 6.5 (0–24)	11.80 ± 6.05 (1–22)	0.815
nonGBA-PD	10.61 ± 7.98 (1–36)	8.38 ± 6.63 (0–34)	7.75 ± 5.68 (0–25)	0.021*

*p*-value (ANCOVA): statistical differences within the GBA-PD and nonGBA-PD groups by age of onset (EOPD, IOPD, LOPD), adjusted for disease duration. Values < 0.05 are marked with an asterisk (*)

*NA* not applicable. *EOPD* early-onset Parkinson’s disease. *IOPD* intermediate-onset Parkinson’s disease. *LOPD* late-onset Parkinson’s disease. *GBA-PD* Parkinson’s disease patients carrying a variant in the *GBA* gene. *nonGBA-PD* Parkinson’s disease patients not carrying a variant in the *GBA* gene. *LEDD* levodopa equivalent daily dose. *MDS-UPDRS* Movement Disorder Society—Unified Parkinson’s Disease Rating Scale. *SCOPA-AUT* Scales for Outcomes in Parkinson’s Disease—Autonomic Dysfunction. *BDI* Beck Depression Inventory

**Table 2:** Cognitive status and MoCA scores across age-at-onset and *GBA1* subgroups

	EOPD	IOPD	LOPD	*p*-value
Raw MoCA score				
GBA-PD	23.97 ± 5.66 (8–30)	23.89 ± 4.68 (11–29)	21.50 ± 2.71 (17–25)	0.045*
NonGBA-PD	24.97 ± 3.04 (17–30)	24.15 ± 3.55 (10–30)	22.24 ± 4.08 (13–30)	< 0.001*
Adjusted MoCA score				
GBA-PD	21.98 ± 5.37 (6.46–28.95)	23.09 ± 4.40 (11.64–29.30)	22.74 ± 3.15 (16.89–26.99)	**0.302**
NonGBA-PD	23.26 ± 2.96 (13.52–27.3)	23.57 ± 2.94 (12.38–28.73)	22.95 ± 3.46 (12.12–28.92)	**0.052**
Normal cognition/MCI/dementia (%)				
GBA-PD	25.6/38.5/35.9	29.6/44.5/25.9	8.3/66.7/25	
NonGBA-PD	19.4/58/22.6 *p*-value: 0.256	20.8/63.4/15.8 *p*-value: 0.199	19.8/58.1/22.1 *p*-value: 0.632	

*p*-value ANCOVA (in bold): Statistical comparisons of continuous outcomes across age-at-onset groups (EOPD, IOPD, LOPD), performed separately within the GBA-PD and PD-GBA cohorts, adjusted for disease duration. *p*-values not in bold are referred to Chi-square test analysis for categorical comparisons between GBA-PD and nonGBA-PD groups, performed within each age-at-onset category (EOPD, IOPD, LOPD). The categorization into normal cognition, mild cognitive impairment (MCI) or dementia was performed for each group by validated cut-off of the adjusted MoCA score. Values < 0.05 are marked with an asterisk (*)

*EOPD* Early-onset Parkinson’s disease. *IOPD* intermediate-onset Parkinson’s disease. *LOPD* late-onset Parkinson’s disease. *GBA-PD* Parkinson’s disease patients carrying a variant in the *GBA* gene. *NonGBA-PD* Parkinson’s disease patients not carrying a variant in the *GBA* gene. *MoCA* Montreal Cognitive Assessment

### GBA-PD and nonGBA-PD patients

After controlling for disease duration, both in GBA-PD and nonGBA-PD groups, cognitive performance did not differ significantly across the three age of onset groups (adjusted MoCA scores—GBA-PD: *p* = 0.302; nonGBA-PD: *p* = 0.052) (Table 1). Early-onset PD (EOPD) patients, in both GBA-PD and nonGBA-PD groups, exhibited significantly more severe motor complications (MDS-UPDRS Part IV scores—GBA-PD: *p* = 0.007; nonGBA-PD: *p* < 0.001). In GBA-PD, EOPD patients also had more severe autonomic symptoms (SCOPA-AUT scores—*p* = 0.012) and a trend toward higher ADL disability (MDS-UPDRS Part II scores—*p* = 0.067); in nonGBA-PD, EOPD showed significantly more severe non-motor symptoms (MDS-UPDRS Parts I scores—*p* < 0.001), higher ADL motor disability (MDS-UPDRS Parts II—*p* < 0.001), more severe depressive symptoms (BDI-II score—*p* = 0.021), and higher LEDD (p < 0.001). Conversely, late-onset PD (LOPD) patients, in both GBA-PD and nonGBA-PD groups, exhibited worse axial symptom scores (GBA-PD: *p* = 0.037; nonGBA-PD: *p* < 0.001).

### The role of age of onset and *GBA1* status

A two-way analysis of variance examined the effects of age of onset, *GBA1* status, and their interaction on clinical outcomes, controlling for disease duration as a covariate (Table 3).

**Table 3:** Impact of age of onset, GBA status, and disease duration on clinical outcomes

Variable	Effect	*F*-value	Partial eta squared	*p*-value
Adjusted MoCA	Model	2.495	0.049	0.023*
	Age of onset	0.508	0.004	0.602
	GBA status	1.249	0.004	0.265
	Disease duration	9.180	0.031	0.003*
	Age of onset * GBA status	0.294	0.002	0.745
MDS-UPDRS Part I	Model	8.334	0.142	< 0.001*
	Age of onset	1.07	0.007	0.344
	GBA status	14.227	0.045	< 0.001*
	Disease duration	15.951	0.05	< 0.001*
	Age of onset * GBA status	1.083	0.007	0.34
MDS-UPDRS Part II	Model	9.527	0.157	< 0.001*
	Age of Onset	1.366	0.009	0.257
	GBA Status	5.591	0.018	0.019*
	Disease Duration	32.283	0.095	< 0.001*
	Age of Onset * GBA Status	1.569	0.01	0.21
MDS-UPDRS Part III	Model	1.879	0.035	0.084
	Age of onset	2.313	0.015	0.101
	GBA status	0.113	0.0	0.737
	Disease duration	8.096	0.026	< 0.005*
	Age of onset * GBA status	0.128	0.001	0.88
MDS-UPDRS Part IV	Model	15.525	0.238	< 0.001*
	Age of onset	6.046	0.039	0.003*
	GBA status	0.083	0.0	0.773
	Disease duration	30.838	0.094	< 0.001*
	Age of onset * GBA status	2.028	0.013	0.133
Axial scores	Model	5.084	0.091	< 0.001*
	Age of onset	3.719	0.024	0.025*
	GBA status	0.087	0.0	0.768
	Disease duration	26.697	0.08	< 0.001*
	Age of onset * GBA Status	0.345	0.002	0.708
BDI-II	Model	3.601	0.074	0.002*
	Age of onset	1.041	0.008	0.354
	GBA status	6.729	0.024	0.010*
	Disease duration	3.199	0.074	0.075
	Age of onset * GBA status	0.110	0.001	0.896
SCOPA-AUT	Model	2.087	0.045	0.055
	Age of onset	0.183	0.001	0.833
	GBA status	0.008	0.0	0.928
	Disease duration	12.111	0.044	< 0.001
	Age of onset * GBA status	0.001	0.0	0.999
LEDD	Model	13.246	0.206	< 0.001*
	Age of onset	1.121	0.007	0.327
	GBA status	0.087	0.0	0.768
	Disease duration	43.386	0.124	< 0.001*
	Age of onset * GBA status	1.053	0.007	0.35

*MoCA* Montreal Cognitive Assessment. *MDS-UPDRS* Movement Disorder Society—Unified Parkinson’s Disease Rating Scale. *BDI* Beck Depression Inventory. *SCOPA-AUT* Scales for Outcomes in Parkinson’s Disease—Autonomic Dysfunction. *LEDD* levodopa equivalent daily dose. *p*-value (ANCOVA): values < 0.05 are marked with an asterisk (*)

Analyzing MoCA scores adjusted for age and education, neither *GBA1* status (*F* = 1.249; *p* = 0.265), nor age of onset (*F* = 0.508; *p* = 0.602) appeared to affect cognition, although GBA-PD patients showed a non-significant trend toward lower scores. Notably, disease duration (used as covariate) significantly influenced MoCA scores.

Age of onset also affected motor complications (MDS-UPDRS part IV scores), which were worse in EOPD (*F* = 6.046; *p* = 0.0013), and axial symptom scores, which were highest in LOPD (F 5.084; *p* < 0.001).

*GBA1* status independently influenced non-motor symptoms and ADL motor disability (MDS-UPDRS parts I and II scores), with GBA-PD patients consistently exhibiting higher scores across all age-of-onset groups (*F* = 14.227; *p* < 0.001 and *F* = 5.591; *p* = 0.019, respectively). Similarly, *GBA1* status significantly impacted depressive symptoms (BDI-II scores), which were higher in GBA-PD patients across all age-of-onset groups (F 6.729; *p* = 0.010).

No significant differences related to age of onset or *GBA1* status were found for motor symptoms (MDS-UPDRS part III), dysautonomic symptoms (SCOPA-AUT), and LEDD (Table 3).

### External cohort (PPMI) validation analysis

MoCA scores were evaluated in an external cohort including 346 PD patients, of whom 309 nonGBA-PD (89.3%) and 37 GBA-PD (10.7%).

Among GBA-PD patients, 43% were EOPD (16/37), 32% IOPD (12/37), and 24% LOPD (9/37). Among nonGBA-PD patients, 16% were EOPD (50/309), 36% IOPD (111/309), and 48% LOPD (148/309). Clinical and demographic data of the PPMI cohort are summarized in Supplementary Table 2.

In the PPMI cohort, age of onset significantly influenced cognitive performances, with late-onset PD showing worse cognitive performances (*F* = 13.579, *p* < 0.001). *GBA1* status and its interaction with age of onset had no significant effect on cognitive decline (Supplementary Table 3).

## Discussion

This study examined a large cohort of over 300 genetically characterized PD patients, including 80 GBA-PD individuals, divided into three groups based on age of onset, allowing a comprehensive assessment of the interplay between genetic status and age of onset on cognitive, motor, and other non-motor outcomes.

To date, it is unknown whether an early age of onset in PD carriers of *GBA1* variants is associated with a worse disease course or whether the younger age is protective against cognitive decline, as observed in the general PD population [1, 17, 33–35].

Using MoCA scores corrected for age and education in a large cohort of patients with an average disease duration of about 7 years, we observed that age of onset, as well as *GBA1* status, did not significantly affect cognitive performances, which was instead significantly driven by disease duration. These results only reflect early to intermediate disease stages and cannot predict cognitive trajectories in later disease phases, where accelerated or non-linear cognitive decline may occur.

Previous literature suggests that, for a comparable disease duration, GBA-PD patients tend to exhibit worse cognitive performance than nonGBA-PD patients. Although *GBA1* carrier status was not a statistically significant determinant of MoCA scores in our cohort (and in the PPMI one), a trend towards lower values was observed in GBA-PD individuals. This finding should be interpreted with caution, as it may reflect limited statistical power rather than true cognitive equivalence between groups, especially considering the cohort’s relatively young age and presumed higher cognitive performance. The PPMI analysis supports our findings; however, disease duration in this cohort was also relatively short [36]. In the PPMI patients, late PD onset was associated with lower MoCA scores, whereas this effect was not observed in our cohort. This discrepancy likely reflects demographic variability, as our sample included fewer late-onset cases and a narrower age range. Another finding of the study is that early age of PD onset in *GBA1* carriers is not associated with greater cognitive impairment within the timeframe evaluated in this study.

These results can be useful for decision-making in clinical practice. This is especially relevant in light of markedly worse motor and non-motor ADL burden observed in GBA-PD patients, along with the more severe motor fluctuations in the early-onset subgroup. Motor and functional impairments represent the most clinically relevant drivers for advanced PD therapies [12–15]. In this context, DBS may represent an important opportunity to improve quality of life in GBA-PD patients while cognition is still relatively preserved, despite the known increased long-term risk of cognitive decline. Importantly, a recent large multicenter study with long-term follow-up suggested that the faster cognitive decline in GBA-PD undergoing DBS is comparable to that observed in non-operated GBA-PD patients, supporting the hypothesis that cognitive decline in GBA-PD undergoing DBS is driven by the genotype itself rather than being exacerbated by DBS [37].

GBA-PD patients may have a greater therapeutic need for DBS because of their heavier motor and non-motor ADL burden and may derive substantial benefit during the years in which they are not yet cognitively deteriorated. Such findings are even more important when considering that GBA-PD patients may have a greater therapeutic need for DBS because of their heavier motor and non-motor ADL burden and may derive substantial benefit during the years in which they are not yet cognitively deteriorated [37].

The finding that dysautonomia severity was not influenced by age of onset nor by *GBA1* status partly contrasts with existing literature. Indeed, a higher frequency and severity of dysautonomic symptoms in GBA-PD has been previously reported [3, 11, 38]. One possible explanation is that the use of SCOPA-AUT as a scale to assess dysautonomic symptom severity in our study was not sensitive enough to catch statistically significant differences between groups with a relatively short disease duration. On the other hand, a recent study using both clinical scales and objective instrumental quantification of dysautonomia showed that while GBA-PD patients reported more symptoms suggestive of orthostatic hypotension, the degree of instrumentally assessed cardiovascular autonomic dysfunction did not differ between *GBA1* carriers and non-carriers [39]. Similarly, a recent study reported that *GBA1* variants do not appear to influence the progression of orthostatic hypotension in PD [40].

Previous literature has highlighted differences between EOPD and LOPD in the general PD population, with EOPD associated with higher burden of motor fluctuations and dyskinesia [17, 18, 41–46], and LOPD associated with worse axial symptoms, such as freezing of gait, postural instability, and gait difficulties [17, 47–49]. Our study confirms these findings both in nonGBA-PD and in GBA-PD patients, showing that age of onset might have a stronger influence than *GBA1* status on the phenotype of patients. This could be explained by a different pathophysiological basis, with faster progression of brain pathology in advanced age, confirmed by a slower disease progression in EOPD compared to LOPD [50], and/or by different brain compensatory mechanisms based on the age of patients [51–54]. Moreover, co-pathology with other neurodegenerative conditions (e.g., beta-amyloid deposition and phosphorylated tau brain accumulation) was frequently observed in older patients [55].

Depression is highly prevalent in PD and is more severe in EOPD [17, 31, 46, 56]. EOPD often face higher psychosocial impact of the disease, with greater challenges related to work, family dynamics, and stigmatization, which can amplify the perception of lost independence and worsen mood. Accordingly, we found higher severity of depressive symptoms and non-motor and motor ADL scores in EOPD nonGBA-PD. In contrast, GBA-PD patients showed consistently higher depression and ADL burden, regardless of age of onset. This finding aligns with previous studies showing that *GBA1* carrier status exacerbates non-motor symptoms, including depression [9, 11, 57]. Conversely, neither age of onset nor *GBA1* status appeared to affect motor symptom severity nor the required dose of dopaminergic therapy.

Despite its strengths, including the multicentric design, the large sample size and the comprehensive evaluation of patients, this study has limitations that need to be considered. These include the cross-sectional design, which precludes analysis of disease progression over time, and the relatively limited disease duration of the included patients. Additional limitations include the exclusive reliance on the MoCA, which lacks sensitivity for detecting domain-specific cognitive impairment; the lack of detailed data on lifetime levodopa exposure as well as the specific use of COMT or MAO-B inhibitors, which precluded modeling the cumulative pharmacological impact on motor and non-motor outcomes; and the absence of stratification by specific *GBA1* variants (prevented by the inadequate sample size to further stratify patients).

Moreover, patients were consecutively enrolled among those who agreed to undergo genetic testing, which could have introduced a selection bias, as this option was likely preferred by younger patients or by those with a family history of PD. In addition, the exclusion of patients receiving advanced therapies was aimed at reducing confounding effects of device-aided interventions on motor and cognitive outcomes but may have led to an underrepresentation of more advanced or severe cases, thus potentially underestimating overall disease burden.

Finally, as data collection occurred across multiple centers, information bias related to inter-site variability in assessments cannot be entirely excluded. Finally, GBA-PD and nonGBA-PD were not balanced for age-group distribution, and age cut-offs were arbitrarily defined.

These limitations notwithstanding, our findings, derived from an original, multicentric cohort and the external PPMI cohort, indicate that within the first 10 years following diagnosis, cognition assessed by MoCA: (1) is significantly determined by disease duration; (2) is not significantly worsened by earlier age of onset even in GBA-PD patients; and (3) is not substantially influenced by *GBA1* mutation carrier status. At the same time, GBA-PD patients already exhibit a heavier motor and functional burden, representing the most immediate determinant for therapeutic planning and a clear marker of unmet clinical needs.

The ability to customize therapeutic strategies based on individual motor and non-motor profiles is essential to improve patients’ quality of life. Future longitudinal studies with systematic recording of treatment trajectories and cumulative levodopa load will be needed to clarify how dopaminergic therapy interacts with age at onset and *GBA1* genotype in shaping disease progression. In addition, larger longitudinal studies using comprehensive neuropsychological batteries will be required to better define the long-term cognitive phenotype associated with *GBA1* variants.

## Supplementary Information

Below is the link to the electronic supplementary material.

Supplementary file1 (DOCX 24 KB)
