# Supplementary material for: The interplay between GBA1 status and age of onset on cognitive, motor and non-motor outcomes in Parkinson’s disease: multicenter cross-sectional study
Source: J Neurol. 2026 Feb 1;273(2):111. doi: 10.1007/s00415-026-13639-x (PMC12862001; doi:10.1007/s00415-026-13639-x)
Supplement: Supplementary file 1 — Supplementary file1 (DOCX 24 KB) [file 415_2026_13639_MOESM1_ESM.docx]

**Supplementary table 1. Study cohort stratified according to age of onset, *GBA* status and *GBA* variant**

| **Age-of-Onset Group** | **GBA-PD (severe)** | **GBA-PD (complex)** | **GBA-PD (mild)** | **GBA-PD (risk)** | **GBA-PD (unknown)** | **Total GBA-PD** | **Total nonGBA-PD** |
| --- | --- | --- | --- | --- | --- | --- | --- |
| **EOPD** | 17  (41%) | 2  (4.8%) | 8  (19.5%) | 9  (21.9%) | 5  (12.2%) | 41  (51%) | 40  (17%) |
| **IOPD** | 11 (40.7%) | 1  (3.7%) | 7  (25.9%) | 5  (18.5%) | 3  (11.1%) | 27  (34%) | 109  (46%) |
| **LOPD** | 2  (16.6%) | 0  (0%) | 2  (16.6%) | 5  (41.6%) | 3  (25%) | 12  (15%) | 87  (36%) |
| **Total** | 30 (37.5%) | 3  (3.8%) | 17 (21.3%) | 19 (23.8%) | 11  (13.8%) | 80  (25%) | 236  (75%) |

EOPD: Early-Onset Parkinson's Disease. IOPD: Intermediate-Onset Parkinson's Disease. LOPD: Late-Onset Parkinson's Disease. GBA-PD: Parkinson's disease patients carrying a variant in the *GBA* gene. nonGBA-PD: Parkinson's disease patients not carrying a variant in the *GBA* gene.

**Supplementary Table 2. Clinical and demographical characteristics of GBA-PD and nonGBA-PD patients stratified by age group in the PPMI cohort**

|  |  | **EOPD** | **IOPD** | **LOPD** | ***p*-value (ANOVA)** |
| --- | --- | --- | --- | --- | --- |
| **Sex**  **%M / %F (M/F)** | GBA-PD | 66.7% / 33,3% (6/3) | 50% / 50% (6/6) | 62.5% / 37.5% (10/6) | 0.704 |
|  | nonGBA-PD | 62% / 38% (31/19) | 60.4% / 39.6% (67/44) | 74.3% / 25.7% (110/38) | 0.041* |
| **Age at evaluation** | GBA-PD | 50.44 ± 5.92 (39-56) | 62.91 ± 3.03 (57-67) | 75.19 ± 5.47 (68-87) | 0.001* |
|  | nonGBA-PD | 51.1 ± 4.94 (39-56) | 62.41 ± 3.35 (57-71) | 74.63 ± 4.82 (68-90) | *<*0.001* |
| **Age of Onset** | GBA-PD | 45.44 ± 5.92 (34-51) | 57.91 ± 3.03 (52-62) | 70.19 ± 5.47 (63-82) | <0.001* |
|  | nonGBA-PD | 46.1 ± 4.94 (34-51) | 57.41 ± 3.35 (52-66) | 69.63 ± 4.82 (63-85) | <0.001* |
| **LEDD** | GBA-PD | 661.43 ± 724.5 (0-2070) | 762.67 ± 665.07 (0-2200) | 370 ± 537.18 (0-1840) | 0.545 |
|  | nonGBA-PD | 1151.56 ± 1079.69 (0-4540) | 681.78 ± 659.05 (0-3784) | 692.26 ± 545.15 (0-3020) | 0.036* |
| **Raw MoCA score** | GBA-PD | 28.78 ± 1.09 (27-30) | 25.08 ± 5.21 (11-30) | 24.38 ± 2.71 (18-30) | 0.025* |
|  | nonGBA-PD | 27.72 ± 2.43 (17-30) | 27.10 ± 2.26 (19-30) | 25.40 ± 3.18 (14-30) | <0.001* |
| **Adjusted MoCA score** | GBA-PD | 26.95 ± 1.22 (24.27-29.43) | 24.29 ± 1.06 (22.14-26.44) | 23.43 ± 0.9 (21.56-25.29) | 0.081 |
|  | nonGBA-PD | 26.05 ± 0.49 (25.08-27.03) | 26.10 ± 0.33 (25.45-26.75) | 24.70 ± 0.29 (24.13-25.26) | 0.002* |

EOPD: Early-Onset Parkinson's Disease. IOPD: Intermediate-Onset Parkinson's Disease. LOPD: Late-Onset Parkinson's Disease. GBA-PD: Parkinson's disease patients carrying a variant in the GBA gene. nonGBA-PD: Parkinson's disease patients not carrying a variant in the *GBA* gene. MoCA: Montreal Cognitive Assessment. LEDD: Levodopa Equivalent Daily Dose. MDS-UPDRS: Movement Disorder Society-Unified Parkinson’s Disease Rating Scale. SCOPA-AUT: Scales for Outcomes in Parkinson's Disease–Autonomic Dysfunction. Values are reported as mean ± standard deviation, with the range of observations given in parentheses (minimum - maximum). *p*-value (ANOVA): Statistical differences within the GBA-PD and nonGBA-PD groups by age of onset (EOPD, IOPD, LOPD). Values < 0.05 are marked with an asterisk (*). NA: not applicable.

**Supplementary Table 3. Impact of age of onset, *GBA* status, and disease duration on MoCA in the PPMI cohort**

| **Variable** | **Effect** | **F-value** | **Partial Eta Squared** | **p-value** |
| --- | --- | --- | --- | --- |
| **Adjusted MoCA** | Model | 3.996 | 0.056 | 0.002* |
|  | Age of Onset | 4.920 | 0.028 | 0.008* |
|  | GBA Status | 1.329 | 0.004 | 0.250 |
|  | Age of Onset * GBA Status | 1.442 | 0.008 | 0.238 |

MoCA: Montreal Cognitive Assessment. MDS-UPDRS: Movement Disorder Society-Unified Parkinson’s Disease Rating Scale. BDI: Beck Depression Inventory. SCOPA-AUT: Scales for Outcomes in Parkinson's Disease–Autonomic Dysfunction. LEDD: Levodopa Equivalent Daily Dose.

p-value (ANCOVA): values < 0.05 are marked with an asterisk (*).
